# Supplementary material for: Conditional chemoconnectomics (cCCTomics) as a strategy for efficient and conditional targeting of chemical transmission
Source: eLife. 2024 Apr 30;12:RP91927. doi: 10.7554/eLife.91927 (PMC11060718; doi:10.7554/eLife.91927)
Supplement: Figure 3—source data 3. [file elife-91927-fig3-data3.zip › Figure 3 source data3 part 2.zip!/Figure 3-Source data 3-part2/1002-9-CRISPResso_Report_e53dd8m5.zip!/CRISPResso_on_1002_10/1c.Alignment_barplot.pdf]

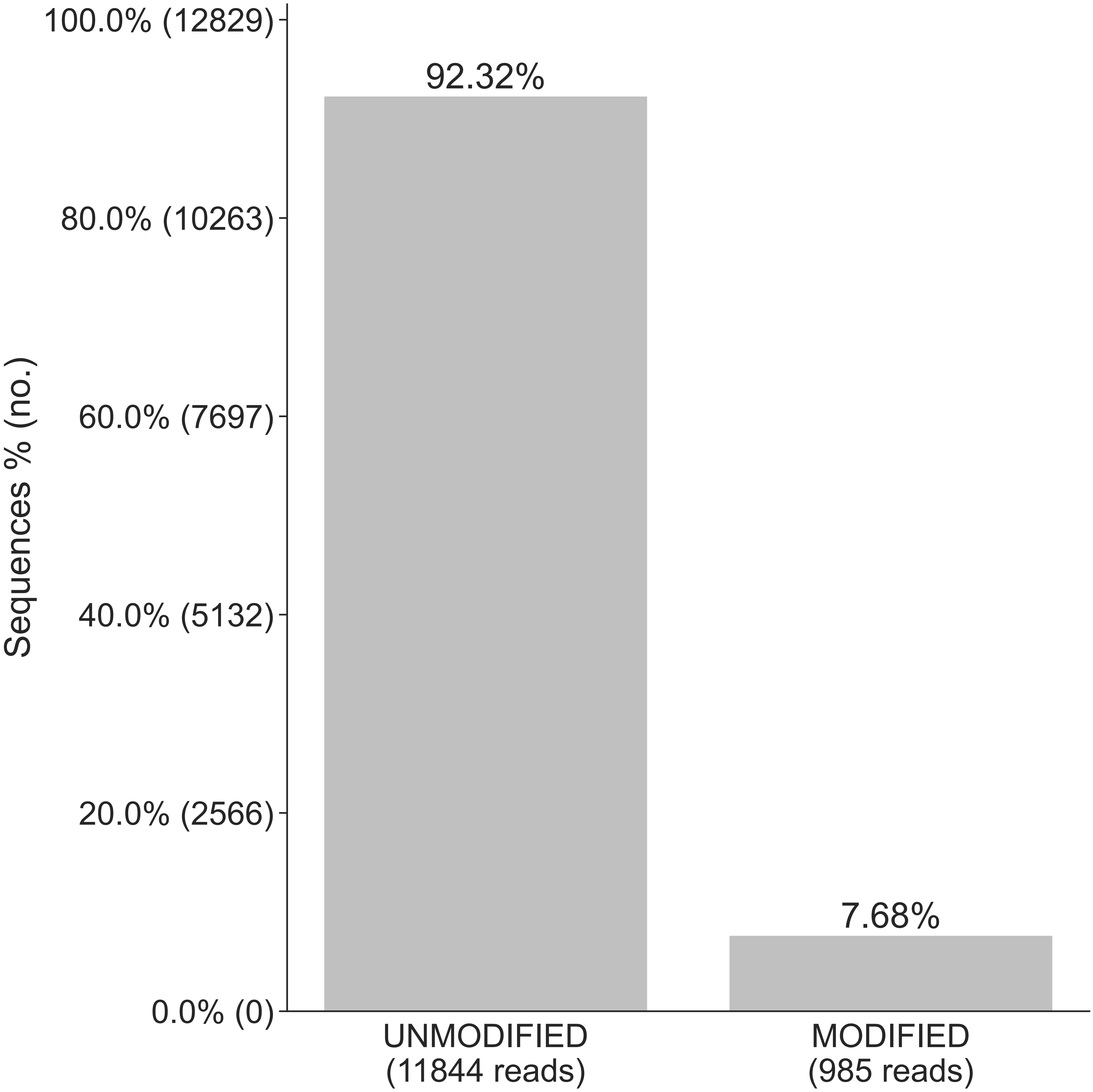
100.0% (12829)
80.0% (10263)
60.0% (7697)
40.0% (5132)
20.0% (2566)
0.0% (0)
Sequences % (no.)
92.32%
7.68%
UNMODIFIED
(11844 reads)
MODIFIED
(985 reads)
